# Supplementary material for: Pannexin-1 Activation by Phosphorylation Is Crucial for Platelet Aggregation and Thrombus Formation
Source: Int J Mol Sci. 2022 May 2;23(9):5059. doi: 10.3390/ijms23095059 (PMC9100471; doi:10.3390/ijms23095059)
Supplement: Supplementary file 1 [file ijms-23-05059-s001.zip › ijms-1681937-supplementary.pdf]

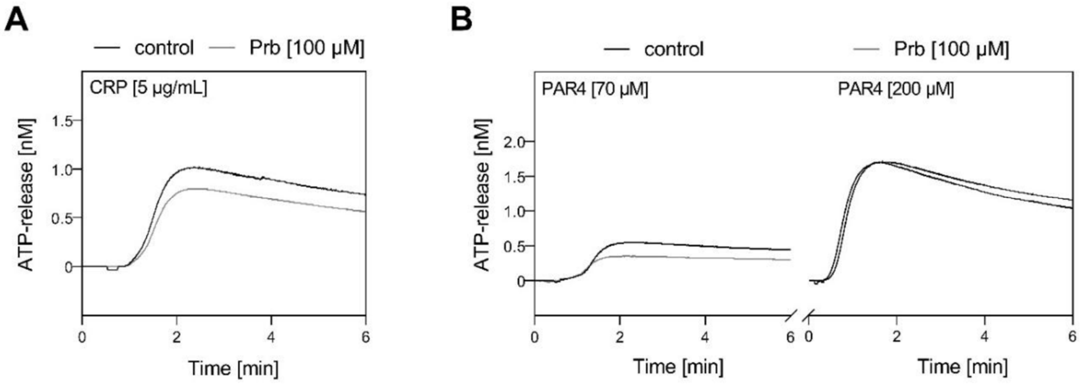

Supplementary Figure S1. Representative curves of ATP release after Probenecid application**.** ATP release was measured with and without 100 µM Prb after platelet activation with (**A**) 5 µg/mL CRP and (**B**) 70 and 200 µM PAR4 peptide via a luciferin/luciferase bioluminescent assay (aggregometer). Curves correspond to Figure 1B. CRP = collagen-related peptide, PAR4 peptide = protease-activated receptor 4 peptide, Prb = probenecid, PANX1 = pannexin-1.

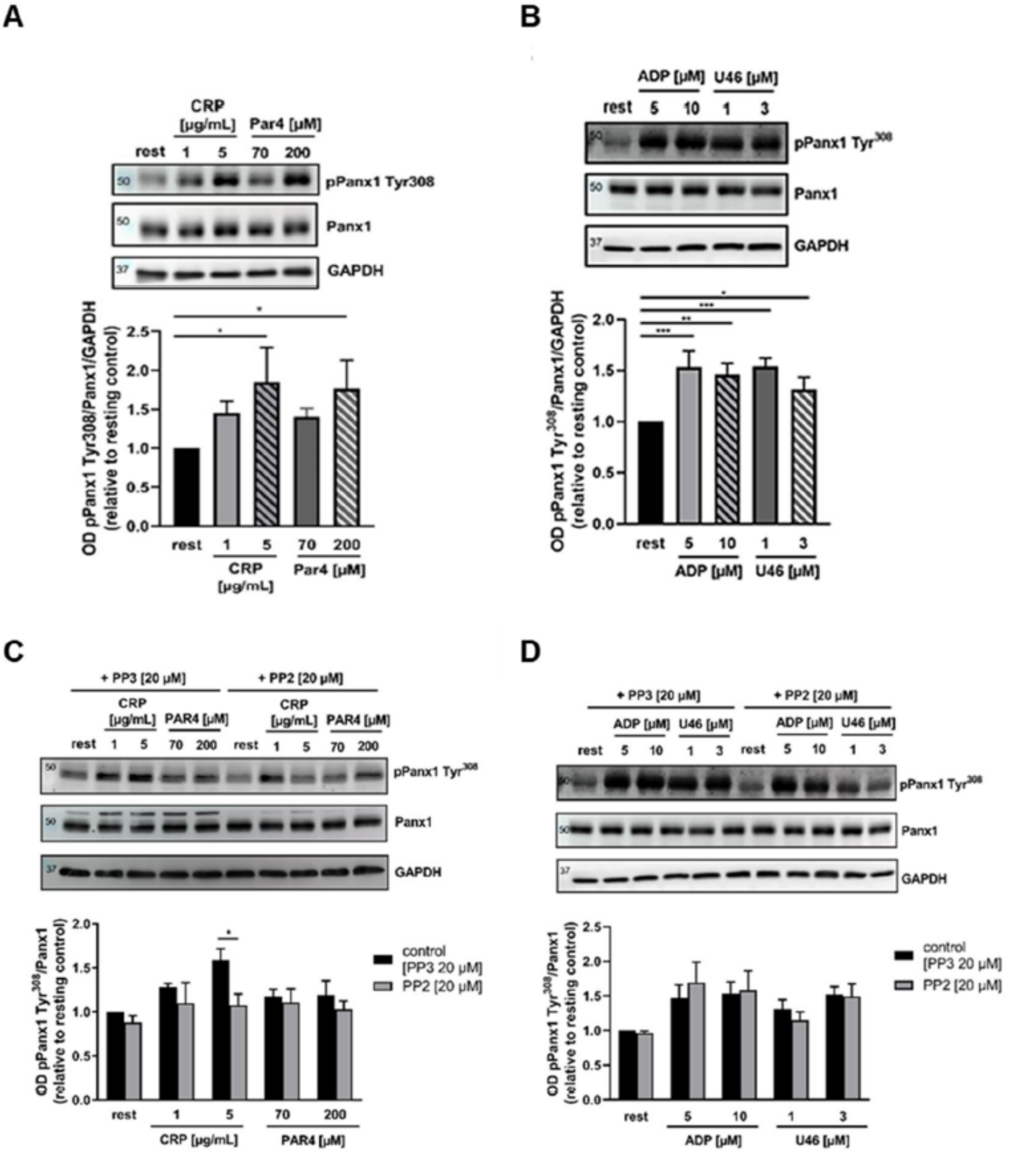

Supplementary Figure S2. PANX1 is phosphorylated at Tyr308 after platelet activation. (**A**–**B**). Representative Western blot images and quantification of phosphorylation of PANX1 at Tyr308 after platelet activation with (**A**) 1 and 5 µg/mL CRP, 70 and 200 µM PAR4 peptide and (**B**) with the second wave mediators ADP and U46619 (n = 4). (**C**–**D**) Western blot analysis and quantification of PANX1 Tyr308 phosphorylation after inhibition of Src family kinases with PP2; PP3 served as a negative control (n = 9). Statistical analyses were performed using a one-way ANOVA (compared to resting) followed by a Sidak's multiple comparisons post-hoc test. Bar graphs indicate mean values ± SEM, *p < 0.05: **p < 0.01 and ***p < 0.001. Rest = Resting, CRP = Collagen-related peptide, PAR4 peptide = Proteaseactivated receptor 4 peptide, U46619 (U46) = Thromboxane analogue, ADP = Adenosine diphosphate, PP2 = SRC kinase inhibitor, PP3 = negative control of PP2.

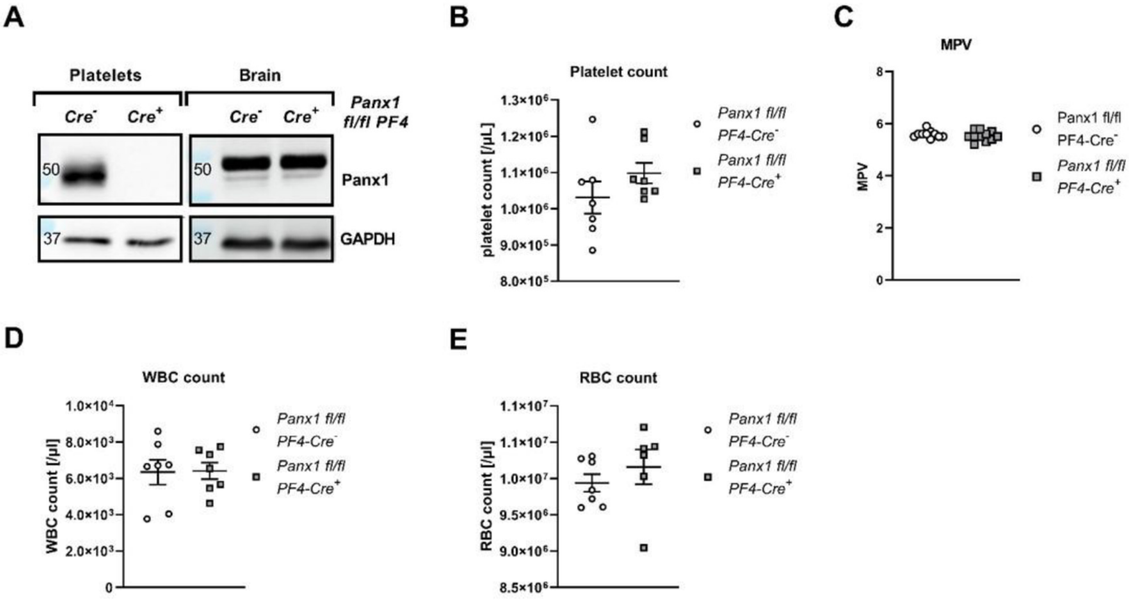

Supplementary Figure S3. Genetic deletion of platelet specific PANX1 does not alter blood cells counts in mice. (**A**) Proof of platelet-specific PANX1 deletion in platelets [1 Mio plts/µL] and brain [50 µg/mL] of *Panx1 fl/fl Pf4-Cre+* and *Panx1 fl/fl Pf4-Cr-*mice. (**B**,**D**,**E**) Blood cell counts of (**B**) platelets, (**D**) WBCs and (**E**) RBCS and (**C**) MPV do not differ between *Panx1 fl/fl Pf4-Cre+* and *Panx1 fl/fl Pf4-Cre-* mice (n=10). Statistical analyses were performed using a two-way ANOVA followed by a Sidak's multiple comparisons post-hoc test. Bar graphs indicate mean values ± SEM, PANX1 = pannexin-1, WBC = white blood cell, RBC = red blood cell, Plts = platelet, MPV = mean platelet volume.

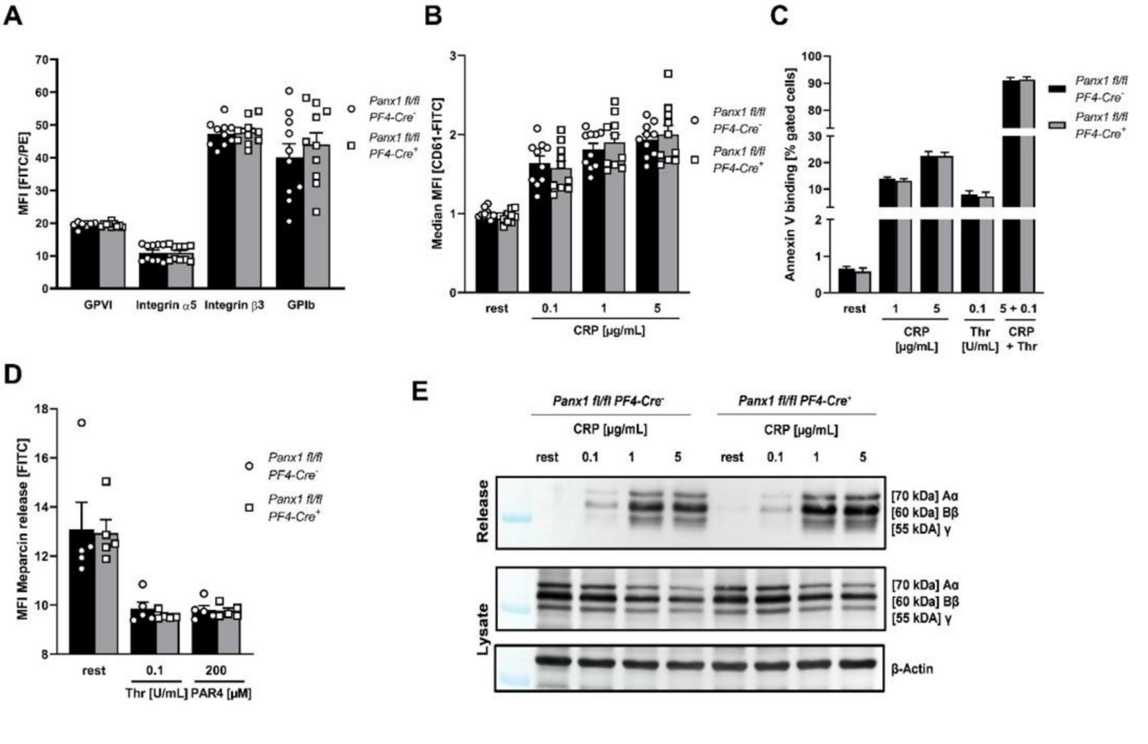

Supplementary Figure S4. Unaltered glycoprotein expression, pro-coagulant activity, and dense granule release of PANX1 deficient platelets. (**A**–**C**) Whole washed blood from *Panx1 fl/fl Pf4-Cre+* and *Panx1 fl/fl Pf4-Cre-* mice was used for flow cytometry. (**A**) Surface expression of glycoproteins was determined (n=10). (**B**) Blood was incubated with indicated concentrations of CRP and CD61 exposure was determined (n=10). (**C**) Annexin V binding of platelets upon stimulation with indicated agonists

was determined (n=5–12). (**D**) Mepacrine release was measured in isolated platelets from *Panx1 fl/fl Pf4-Cre+* and *Panx1 fl/fl Pf4-Cre-* mice using flow cytometry (n=5). (**E**) Fibrinogen release was analyzed using isolated platelets from *Panx1 fl/fl Pf4-Cre+* and *Panx1 fl/fl Pf4-Cre-* mice by immunoblotting (n=3). Statistical analyses were performed using a two-way ANOVA followed by a Sidak's multiple comparisons post-hoc test. Bar graphs indicate mean values ± SEM. Rest = resting, CRP = collagen related peptide, Par4 peptide = protease-activated receptor 4 peptide, Thr = thrombin, PANX1 = pannexin-1.

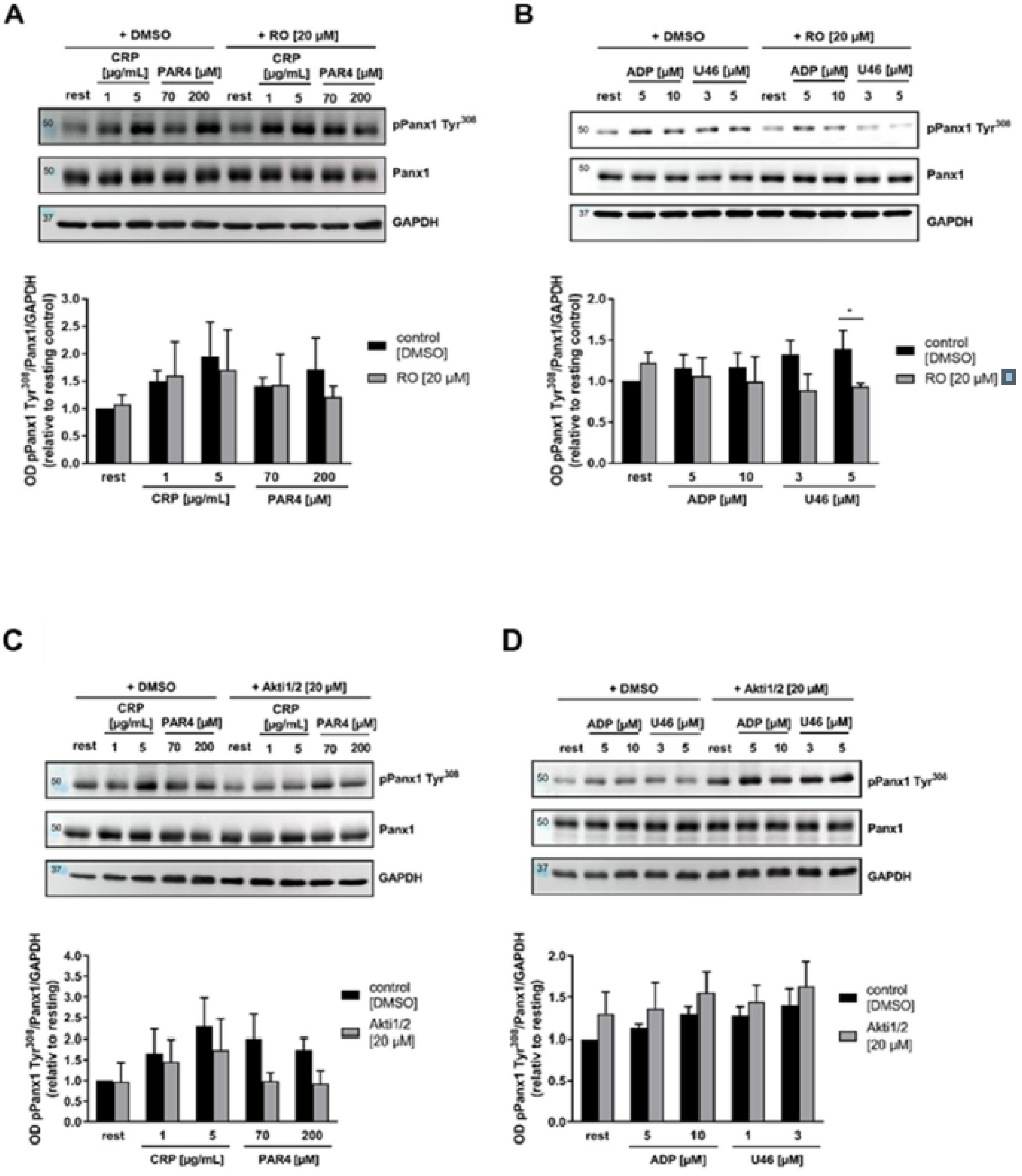

Supplementary Figure S5. Inhibition of PKC by RO leads to decreased phosphorylation of PANX1 at Tyr$_{308}$ upon platelet activation with high dose of U46619. Representative Western blot images and quantification of phosphorylation of PANX1 at Tyr$_{308}$ after inhibition of PKC by pre-incubation of platelets with Ro-31-8220 (RO) or DMSO as negative control. (**A**) Platelets were activated with 1 and 5µg/mL CRP and 70 and 200 µM PAR4 peptide (n = 3). (**B**) Platelets were activated with 5 and 10 µMADP and 1 and 3 µM U46619 (n = 3). (C-D) Representative Western blots and quantification of PANX1 phosphorylation at Tyr$_{308}$ after inhibition of Akt by pre-incubation of platelets with 20 µM

Akti1/2 or DMSO as negative control. (**C**) Platelets were activated with 1 and 5 µg/mL CRP, 70 and 200 µM PAR4 peptide (n = 3) or (**D**) with 5 and 10 µM ADP and 1 and 3 µM U46619 (n = 5). Statistical analyses were performed using a two-way ANOVA followed by a Sidak's multiple comparisons post-hoc test. Bargraphs indicate mean values ± SEM, **p* < 0.05: ***p* < 0.01 and ****p* < 0.001. Rest = Resting, CRP = Collagenrelated peptide, PAR4 peptide = Protease-activated receptor 4 peptide, ADP = Adenosine diphosphate, U46 (U46619) = Thromboxane A2 analogue, PKC = Protein Kinase C; DMSO = Dimethyl sulfoxide, Akti1/2 = Akt-inhibitor 1/2.

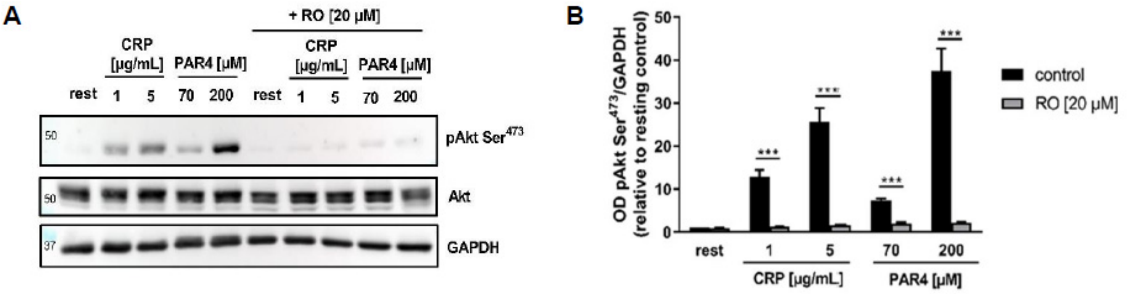

Supplementary Figure S6. Inhibition of PKC leads to reduced Akt phosphorylation at Ser$_{473}$ after platelet activation with PAR4 peptide. (**A**) Representative immunoblot and (**B**) quantification of phosphorylation of Akt at Ser$_{473}$ after inhibition of PKC by pre-incubation of platelets with Ro-31-8220 (RO) or DMSO as a negative control. Platelets were activated with 1 or 5 µg/mL CRP and 70 or 200 µM Par4 (n=3). Statistical analyses were performed using a two-way ANOVA followed by a Sidak's multiple comparisons post-hoc test. Bar graphs depict mean values ± SEM, ****p* < 0.001. Rest = resting, CRP = collagen-related peptide, PAR4 peptide = protease-activated receptor 4 peptide, ADP = adenosine diphosphate, PKC = protein kinase C, DMSO = dimethyl sulfoxide, PANX1 = pannexin-1.

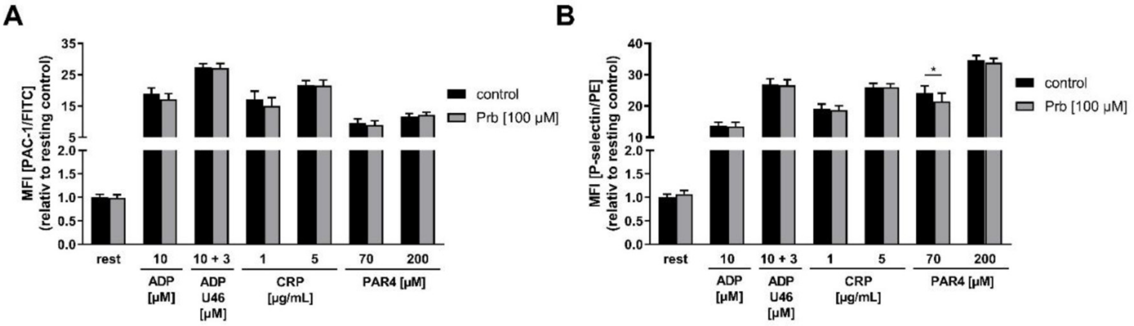

Supplementary Figure S7. Pharmacological inhibition of PANX1 alters degranulation following platelet stimulation with low dose of PAR4 peptide. (**A**,**B**) Human platelets were isolated, pre-incubated with the PANX1 inhibitor Prb and activated with standard agonists as indicated. (**A**) Integrin $\alpha_{IIb}\beta_3$ and (**B**) P-selectin exposure were measured by flow cytometry (n=10-14). Statistical analysis was performed using a mixed-effect analysis followed by a Sidak's multiple-comparison post-hoc test. Bar graphs indicate mean values ± SEM, **p* < 0.05: ***p* < 0.01 and ****p* < 0.001. Rest = resting, ADP = adenosine diphosphate, CRP = collagen-related peptide, PAR4 peptide = protease-activated receptor 4 peptide, U46619 (U46) = thromboxane analog, Prb = probenecid.
